# The Bacterial DNA Profiling of Chorionic Villi and Amniotic Fluids Reveals Overlaps with Maternal Oral, Vaginal, and Gut Microbiomes

**DOI:** 10.3390/ijms24032873

**Published:** 2023-02-02

**Authors:** Giuseppina Campisciano, Nunzia Zanotta, Mariachiara Quadrifoglio, Annalisa Careri, Alessandra Torresani, Carolina Cason, Francesco De Seta, Giuseppe Ricci, Manola Comar, Tamara Stampalija

**Affiliations:** 1 Department of Advanced Translational Microbiology, Institute for Maternal and Child Health—IRCCS Burlo Garofolo, Via dell’Istria, 65, 34137 Trieste, Italy; 2 Unit of Fetal Medicine and Prenatal Diagnosis, Institute for Maternal and Child Health—IRCCS Burlo Garofolo, Via dell’Istria, 65, 34137 Trieste, Italy; 3 Department of Medicine, Surgery and Health Sciences, University of Trieste, Strada di Fiume 447, 34149 Trieste, Italy; 4 Department of Obstetrics and Gynecology, Institute for Maternal and Child Health—IRCCS Burlo Garofolo, Via dell’Istria, 65, 34137 Trieste, Italy

**Keywords:** maternal-fetal microbiota axis, in utero microbiome, sterile womb hypothesis, pregnancy, chorionic villi, amniotic fluid

## Abstract

The in utero microbiome hypothesis has been long debated. This hypothesis will change our comprehension of the pioneer human microbiome if proved correct. In 60 uncomplicated pregnancies, we profiled the microbiome of chorionic villi (CV) and amniotic fluids (AF) in relation to maternal saliva, rectum, and vagina and the soluble cytokines cascade in the vagina, CV and AF. In our series, 12/37 (32%) AF and 10/23 (44%) CV tested positive for bacterial DNA. CV and AF harbored bacterial DNA of Streptococcus and Lactobacillus, overlapping that of the matched oral and vaginal niches, which showed a dysbiotic microbiome. In these pregnant women, the immune profiling revealed an immune hyporesponsiveness in the vagina and a high intraamniotic concentration of inflammatory cytokines. To understand the eventual role of bacterial colonization of the CV and AF and the associated immune response in the pregnancy outcome, further appropriate studies are needed. In this context, further studies should highlight if the hematogenous route could justify the spread of bacterial DNA from the oral microbiome to the placenta and if vaginal dysbiosis could favor the likelihood of identifying CV and AF positive for bacterial DNA.

## 1. Introduction

The host–microbiome interrelationship is considered a mutualistic symbiosis in which the human body provides sustenance and an adequate environment for the microbial growth, while the microbes accomplish essential functions, such as helping the immune system develop, metabolic functions, and defense against infections [1].

The in utero microbiome has been debated for almost 150 years [2,3]. According to the sterile womb paradigm, microbes are acquired both vertically (from the mother) and horizontally (from other humans or the environment) during and after birth whilst the fetus is maintained in a sterile state [4]. Oppositely, we recently identified bacterial colonization of placenta and amniotic fluid during early prenatal life [5]. These results are in line with some other recent studies exploiting the high-throughput sequencing technologies that have challenged this paradigm, proposing that neither the fetus, the placenta, nor the amniotic fluid are sterile and that the acquisition of microbes begins in utero [6,7,8]. At first, the theory of the non-sterile uterus was explained by underlying contamination issues. Indeed, it is now broadly recognized that laboratory reagents harbor “per se” low levels of bacterial DNA [9]. While this contamination does not affect studies including highly colonized samples, e.g., stool samples, it becomes a major issue when working with low microbial biomass samples, e.g., amniotic fluids. Accurate and controlled experimental protocols, including the removing of contamination from sequencing reagents (the “kitome”), thanks to the denoising methods, are able to minimize contamination in the microbiome workflow. Denoising methods are based on an error model that takes into account the quality of the sequencing run, distinguishing between the predicted “true” biological variation and the variation likely generated by sequencing errors, identifying real biological sequences at a single nucleotide resolution by producing amplicon sequence variants (ASVs) [10,11,12,13].

If this “in utero colonization hypothesis” proves correct, our comprehension of the establishment of the pioneer human microbiome and its relationship with environmental, lifestyle, and clinical factors will change. Indeed, prenatal microbial colonization is expected to exert a significant influence on the developing fetus, e.g., through the production of short chain fatty acids (SCFAs), positively affecting the immune system’s development [14] or, conversely, by the immune sensitization resulting in the production of a plethora of inflammatory mediators [15,16].

Thus, hypothesizing that the microbial footprint and its metabolic and immunological priming of the newborn happen already in utero during the first phase of pregnancy, it is important to understand how the microbial transfer occurs. Although the vaginal microbiome plays a potential role in successful fertilization and healthy pregnancies [17], the presence of bacterial strains in the other districts of the reproductive tract suggests another possible source of fetal bacterial colonization than the vaginal microbiome.

In order to investigate the origin of the fetal-placental microbiome during the early phases of pregnancy, we have investigated the bacterial composition of chorionic villi and amniotic fluids in relation to maternal saliva, the rectum, and the vagina during the first and second trimesters of gestation. Next, in order to explore how the maternal-fetal microbiome transfer may impact immune tolerance, we dosed the soluble cytokine cascade in vaginal, chorionic villi, and amniotic fluid samples.

## 2. Results

In the present study, 64 Caucasian women carrying singleton pregnancies were included, with a mean age of 38 ± 4 years. The indications for invasive procedures were fetal malformation (21/64, 32.8%), advanced maternal age (22/64, 34.4%), high-risk screening test (8/64, 12.5%), anamnestic risk (8/64, 12.5%), combined advanced maternal age and high-risk screening test (2/64, 3.1%), combined advanced maternal age and fetal malformation (1/64, 1.6%), and combined advanced maternal age and anamnestic risk (2/64, 3.1%). Before further analysis, four women were excluded from the study as the chorionic villus/amniotic fluid sampling was not sufficient for downstream analysis.

A total of 240 biological samples were sequenced, including chorionic villi or amniotic fluid, vaginal swabs, rectal swabs, and saliva samples from 60 pregnant women.

After the DADA2 filtering, the sequencing of the V3 region of the 16S rRNA gene produced a total of 9,916,147 reads, identifying 15,207 features. The 24 no template controls produced a total of 59,290 reads, identifying 1560 features.

For the analysis, we grouped the samples as follows: chorionic villi samples (CVS, n = 23) and the matched samples, including vaginal swabs (Vag.CVS), rectal swabs (Rect.CVS), and saliva samples (Sal.CVS). The same sample grouping was performed for the amniotic fluid samples (AF, n = 37) and matched samples, including vaginal swabs (Vag.AF), rectal swabs (Rect.AF), and saliva samples (Sal.AF).

We computed the core microbiome of the no template controls, identifying features present in at least 30% of these samples, including *Ralstonia pickettii*, *Escherichia coli*, *Bacillus pseudofirmus*, *Cutibacterium acnes,* and *Pseudomonas fulva* sequences. There were no shared features when considering more than 30% of the no template controls. Table S1 shows the unique bacteria identified in one or more no template controls and that were identified in less than 30% of these samples. All the bacteria identified in the no template controls were filtered out from the taxonomic assignment results of the biological samples. Thus, the CVS and AF samples showing no differences in the identified features with no template controls were considered negative in the present analysis. After the sorting, 12/37 (32%) AF samples and 10/23 (44%) CVS samples tested positive for the presence of bacterial DNA.

**Figure 1:**
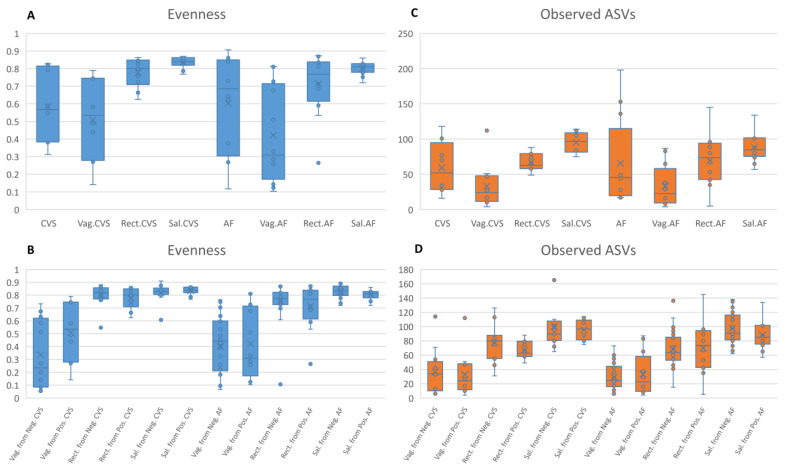
(**A**,**B**) Alpha diversity in AF and CVS samples. The alpha diversity metrics measured by means of the evenness and observed ASVs metrics. CVS = chorionic villus samples; Vag.CVS = vaginal swabs matched to CVS; Rect.CVS = rectal swabs matched to CVS samples; Sal.CVS = saliva samples matched to CVS; AF = amniotic fluid samples; Vag.AF = vaginal swabs matched to AF; Rect.AF = rectal swabs matched to AF samples; Sal.AF = saliva samples matched to AF. The comparisons among groups were performed by the Kruskal–Wallis test. (**C**,**D**) Alpha diversity in different body sites. The alpha diversity metrics measured by means of the evenness and observed ASVs metrics. The comparisons among groups were performed by the Kruskal–Wallis test.

Including the positive samples and retaining 5000 reads per sample, we tested the alpha diversity by the evenness and observed ASV metrics. Comparing these samples with the matched vaginal, rectal, and saliva samples, only one significant difference was observed for the evenness metric (CVS vs. Sal.CVS, Kruskal–Wallis test, FDR *p* value = 0.008) (Figure 1A). When comparing the vaginal, rectal, and saliva samples matched to the positive CVS/AF samples with the vaginal, rectal, and saliva samples matched to the negative CVS/AF samples, there were no significant differences (Figure 1C,D).

**Figure 2:**
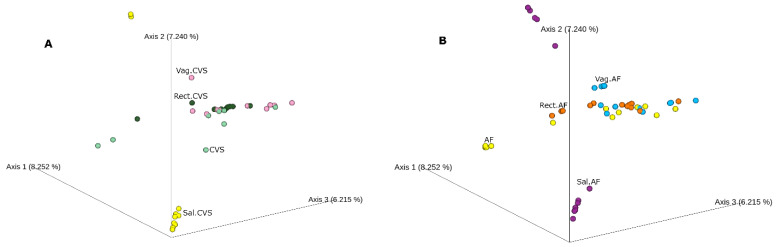
The beta diversity. Principal coordinate analysis (PcoA) based on Bray–Curtis dissimilarity matrix of bacterial communities in the analyzed groups. (**A**) CVS positive for bacterial DNA (light green) and matched vaginal (pink), rectal (dark green), and saliva (yellow) samples. (**B**) AF samples (yellow) and matched vaginal (light blue), rectal (orange), and saliva (purple) samples. Not all the analyzed samples are visible; hiding samples in an ordination can be misleading.

After that, we performed a beta diversity analysis. Figure 2 shows that both CVS and AF clustered near the vaginal and rectal samples. Regardless of the graphical clustering, the pairwise PERMANOVA highlighted significant differences in these sample groups (Table S2).

At this point, we performed the differential abundance testing using the ANCOM test. The bacteria that significantly changed between the compared groups were *Anaerococcus* (W = 551), *Corynebacterium* (W = 560), *Dialister* (W = 561), *Gemella* (W = 554), *Haemophilus* (W = 556), *Lactobacillus* (W = 561), *Mobiluncus* (W = 520), *Peptoniphilus* (W = 552), *Porphyromonas* (W = 561), *Prevotella* (W = 561), *Streptococcus* (W = 560), *Staphylococcus* (W = 543), and *Veillonella* (W = 558).

**Figure 3:**
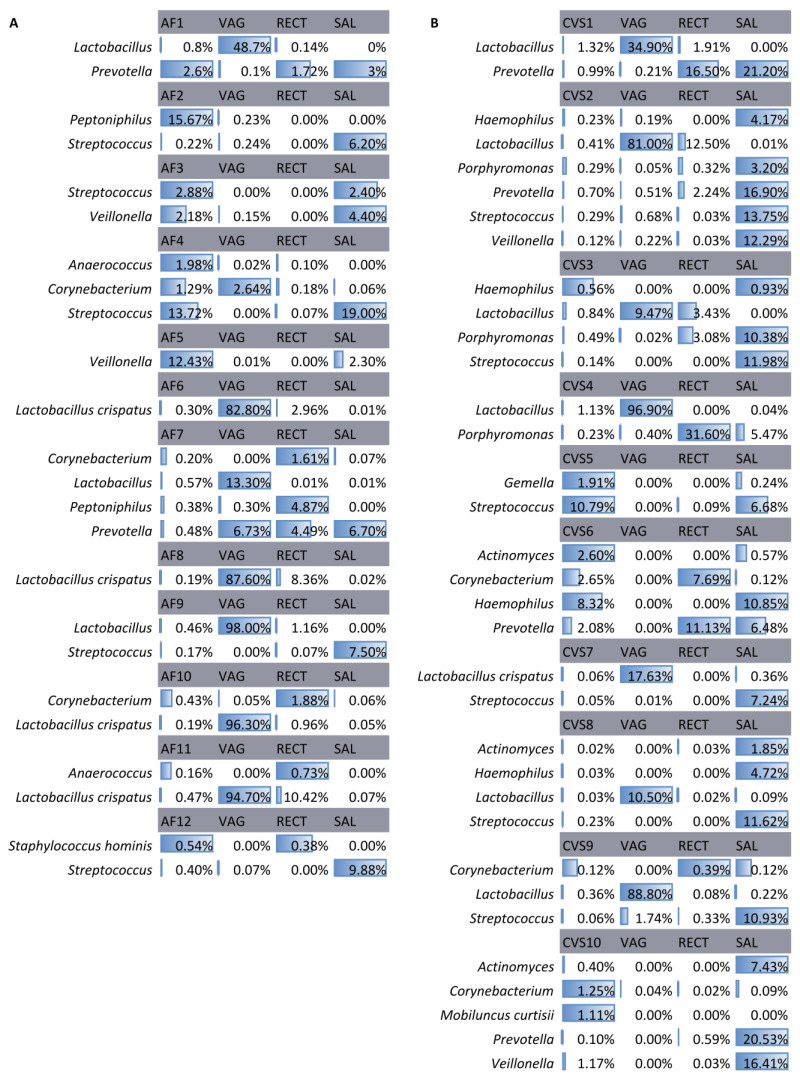
Bacteria in positive AF samples (**A**) and CVS (**B**) and their matched samples. The identified bacteria in the amniotic fluid (AF) samples and chorionic villi samples (CVS) were positive for the presence of bacterial DNA and in the matched vaginal (VAG), rectal (RECT), and saliva (SAL) samples. Data are presented as relative abundances.

In AF-positive samples, the most frequently identified bacterial DNA belonged to *Lactobacillus* (n = 5), which was shared with the vaginal samples, and *Streptococcus* (n = 5), which was shared with the saliva samples (Figure 3). A higher microbial heterogeneity was observed in positive CVS, in which the most frequently identified DNA belonged to *Lactobacillus* (n = 7), shared with vaginal samples, and *Streptococcus* (n = 6), shared with vaginal and saliva samples (Figure 3). 

Then, we applied the LEfSE test to identify microbiological biomarkers in the vaginal, rectal, and saliva samples matched to the negative CVS/AF samples compared with those matched to the positive CVS/AF samples (Table S3).

**Figure 4:**
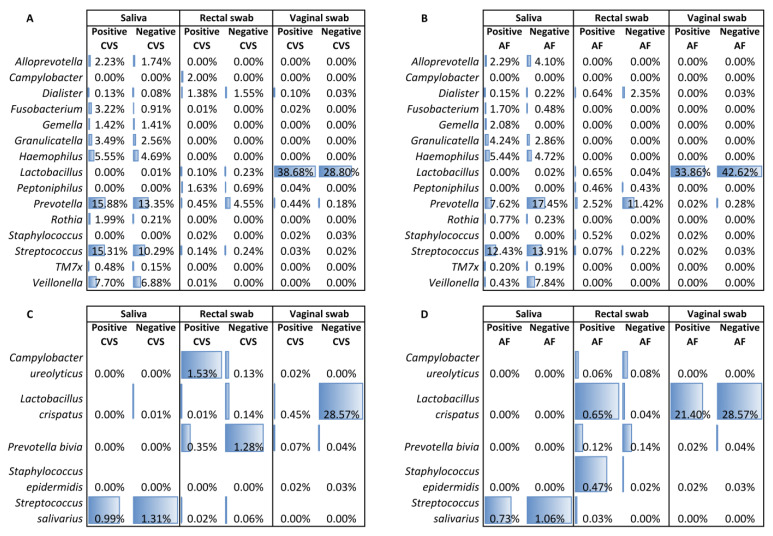
(**A**,**B**) Significantly different bacterial genera among samples matched to positive and negative CVS and AF samples. (**C**,**D**) Significantly different bacterial species among samples matched to positive and negative CVS and AF samples. Biomarkers identified by the LEfSe test. Data are shown as median relative abundances.

The saliva samples that matched the positive CVS and the positive AF samples showed a significantly increased abundance of several bacterial genera (e.g., *Fusobacterium*, *Granulicatella*, *Haemophilus*, *Prevotella*, *Streptococcus,* and *Veillonella*) compared to those matched to negative CVS/AF samples. Concerning the rectal samples, those matched to the positive CVS and AF samples showed a higher relative abundance of *Campylobacter*, *Peptoniphilus*, and *Lactobacillus*. In the vaginal samples matched to positive CVS, an increase in lactobacilli was observed compared to the vaginal samples matched to negative CVS, whereas the opposite trend was observed for the vaginal samples matched to positive AF samples (Figure 4A,B).

The same analysis was performed at the species level (Table S4). 

Figure 4C shows the increase in *S. salivarius* in the saliva samples and of *L. crispatus* in the vaginal samples matched to the negative CVS when compared to the samples matched to the positive CVS. *C. ureolyticus* increased in the rectal samples matched to the positive CVS, while in these samples, *P. bivia* decreased compared to the rectal samples matched to the negative CVS.

Figure 4D revealed a similar trend to that observed for the saliva and vaginal samples matched to the CVS concerning *S. salivarius* and *L. crispatus*. With regard to the rectal samples, *L. crispatus*, *S. epidermidis,* and *S. salivarius* increased in those samples matched to the positive AF samples compared to those matched to the negative AF samples.

**Figure 5:**
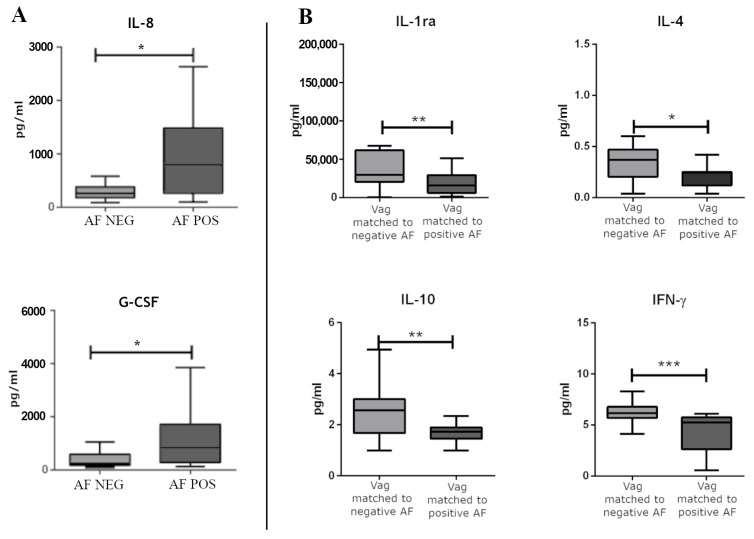
(**A**) The significantly modulated immune soluble factors between AF samples positive for bacterial DNA and AF samples negative for bacterial DNA. (**B**) The significantly modulated immune soluble factors between vaginal swabs matched to negative and positive AF samples for bacterial DNA. Differences were calculated by means of a non-parametric T test for pairwise comparisons (GraphPad Prism v. 5). * *p* < 0.05, ** *p* < 0.01, *** *p* < 0.001.

Concerning the local immune response, we did not observe significantly modulated immune factors when comparing positive CVS to negative CVS. In regard to AF-positive samples, IL-8 and granulocyte colony-stimulating factors (G-CSF) were significantly upregulated in AF samples positive for bacterial DNA compared with the AF samples negative for bacterial DNA (Figure 5A).

When accounting for the immune-soluble factors dosed in the vaginal swabs, we did not observe significant differences in the vaginal swabs matching the CVS. On the contrary, we observed significantly modulated factors in vaginal swabs matched to the AF samples positive for the bacterial DNA compared to vaginal swabs matched to the AF samples negative for the bacterial DNA. Namely, IL-4, IL-10, IFN-γ, and IL-1ra were significantly down-regulated in vaginal swabs matched to the AF samples positive for the bacterial DNA (Figure 5B).

## 3. Discussion

In the present study, we examine in utero colonization by analyzing the microbiome of chorionic villi and the amniotic fluids of 60 singleton pregnancies. In addition, in order to identify the possible origin of the identified bacterial DNA, vaginal, rectal, and saliva samples collected at the same time as CVS or AF sampling were tested.

In the CVS and AF samples positive for bacterial DNA, a heterogeneous microbiome was observed, similar to that of the matched vaginal, rectal, and saliva samples (Figure 1). In particular, in the vaginal samples matched to the positive CVS, a slightly uneven distribution of the microbial relative abundances was evident, suggesting the presence of the vaginal commensal *Lactobacillus* spp. alongside several other bacteria [18].

Apart from the heterogeneity of the microbial community, positive CVS showed a partial overlap in terms of bacterial identity with the matched vaginal and rectal samples. We can speculate that only some bacterial species are shared between these body districts. The same observation was confirmed for the AF-positive samples, which clustered near vaginal and rectal samples (Figure 2).

When accounting for the significantly modulated bacteria, several bacteria were identified. The identified bacteria in the CVS and AF-positive samples belonged to commensal and opportunistic pathogens of the reproductive tract and the oral cavity [18,19] that were not identified in the negative samples. This identification was consistent with the presence of these bacteria in one or more matched samples. Our results show that CVS harbors greater microbial heterogeneity, in particular regarding the possibly derived oral species. The previously formulated hypothesis of a hematogenous route or low-grade bacteremia would justify the bacterial DNA colonization of the placenta from the oral microbiome [20]. In this regard, the amniotic fluid, being less exposed to the hematogenous access, would be less prone to colonization by oral microbes (Figure 3).

We observed that in saliva samples matched to the positive CVS and, to a minor extent, in the oral microbiome of the colonized AF samples, *Fusobacterium*, *Prevotella*, *Streptococcus,* and *Veillonella* increased compared to the saliva samples matched to the negative CVS/AF samples. During pregnancy, an increase in these opportunistic pathogens [21,22,23] and a decrease in probiotic strains able to inhibit immune activation by periodontal disease pathogens, such as *Streptococcus salivarius*, have been previously observed [24,25] In our cohort, *Streptococcus salivarius* decreased in saliva samples matched to positive CVS/AF samples in concomitance with the increase in keystone low-abundance microbial pathogens, remodeling a normally eubiotic microbiota into a dysbiotic one [26,27]. Although not demonstrable by our data, it is noteworthy that the administration of a probiotic *Streptococcus salivarius* strain has been suggested as a preemptive treatment to limit vaginal colonization from pathogens during pregnancy and thereby prevent neonatal transmission [28,29]. Based on the hypothesis of low-grade bacteremia, oral dysbiosis should be studied to reveal if it triggers the bacterial DNA colonization of the placenta from the oral niche.

In vaginal samples, the most evident result was the absence and decrease, respectively, of *L. crispatus* in the samples matched to the positive CVS and AF samples compared to the samples matched to the negative CVS/AF samples. To note, the association of *L. crispatus* with the stability of the vaginal microbiota has been observed, especially during pregnancy [30,31] (Figure 4). As was observed for *S. salivarius*, *L. crispatus* has long been studied for its role in protecting the vaginal epithelium from pathogen colonization, such as *Streptococcus agalactiae*, which predisposes to a number of adverse pregnancy outcomes, including stillbirth, preterm birth, and neonatal invasive disease [32].

The bacterial DNA translocation could be elicited or accompanied by an altered local immune response. Our results support the presence of significant differences between the AF samples positive for the presence of bacterial DNA and the AF samples negative for the presence of bacterial DNA. In particular, markers of intraamniotic inflammation, such as IL-8 and G-CSF, were increased in the presence of bacterial DNA in the AF samples [33,34,35]. To note, the increment of IL-8 in the AF-positive samples was below the cutoff described in scientific literature as being associated with adverse outcomes, likely justifying the absence of a manifest inflammation in our series [35,36,37]. These factors were not significantly modulated in the matched vaginal swabs, and there were no differences between the positive and negative CVS and their matched vaginal swabs, confirming that the mother, placenta, and fetus all possess unique innate immune systems [38]. In this series, IL-4, IL-10, IFN-γ, and IL-1ra were found downregulated in vaginal swabs matched to positive AF samples. To note, an increase in Th2 cytokines, including IL-4, IL-10, and IL-1ra are fundamental for promoting a healthy, successful pregnancy by suppressing inflammation [39,40,41], while IFN-γ plays critical roles in the activation of innate and adaptive immune responses to pathogens. Alterations in these processes are believed to contribute to gestational complications [42,43]. We can speculate that immune hyporesponsiveness, represented by low cervicovaginal concentrations of various proinflammatory cytokines and high intraamniotic concentrations of proinflammatory cytokines, could be further studied to understand if it is a risk factor for bacterial DNA translocation among women with lower genital tracts with altered microbial composition [44] (Figure 5).

## 4. Material and Methods

### 4.1. Study Design

This is a prospective longitudinal single-center study in which women with singleton pregnancy afferent to Fetal Medicine and Prenatal Diagnosis of the IRCCS Burlo Garofolo hospital in Trieste, Italy, for the execution of chorionic villus sampling (first trimester of pregnancy) or amniocentesis (second trimester of pregnancy) were enrolled. The study was proposed to patients who decided to perform an invasive diagnostic procedure for clinical reasons, such as advanced age, previous medical history (genetic pathology), or a high-risk screening test for major aneuploidies. Women with sexually transmitted infections, hormonal or antibiotic/probiotic therapy in the previous 6 months to the enrollment, history of chronic or infectious diseases, history of recurrent vaginal and urinary infections, or documented risk factors (smoking, obesity, or drug use) were excluded. All women were asymptomatic at the time of the invasive procedure. All enrolled patients had to give their consent to use excessive material for the purposes of the study.

In this study, 60 pregnant women were enrolled. Chorionic villi or amniotic fluid were collected from each pregnant woman. In addition, swabs were performed in vaginal, oral, and rectal maternal body districts.

### 4.2. Biological Sampling Procedures

Chorionic villus sampling is an invasive procedure performed in our center in the first trimester (11–14 weeks) trans-abdominally, after preparation of a sterile field, and involves the insertion of a guide needle of 18 G inside the placenta. Subsequently, the second 20-G needle is introduced, with which the villi are aspirated. The quantification of the sampled villi is performed only at the end of the procedure. Approximately 15 mg of villi are sufficient for cytogenetic analysis. Cytogenetic analysis was privileged only in the case in which there was an excess quantity of the collected villi; these were destined for the study and were deposited in a sterile container with 1 mL of sterile physiological solution and immediately sent to the laboratory of Advanced Microbiological Translational Diagnostics.

Amniocentesis is an invasive procedure performed in our center starting from the 15th week of gestation and is carried out trans-abdominally. After the preparation of a sterile field, amniocentesis involves the insertion of a single 21-G needle. Usually, 16–20 cc of liquid is withdrawn for cytogenetic analysis, depending on gestational age. For the purpose of the study, we used 1–2 cc of amniotic fluid that was deposited in a sterile container and immediately sent to the laboratory of Advanced Microbiological Translational Diagnostics.

The saliva samples were collected in an empty sterile tube and harvested at least 1 h after the last meal and after subsequent oral hygiene. The rectal swabs (APTACA Spa, Regione Monforte, Canelli, Italy) were obtained by inserting 1–3 cm to ensure sampling of the distal rectum. The vaginal swabs (APTACA Spa, Regione Monforte, Canelli, Italy) were obtained by a single gentle 360° rotation at the vaginal wall under speculum examination. Amniotic fluid and chorionic villi samples were stored at −80 °C until the time of analysis. Regarding the swabs, they were suspended in 1.5 mL of sterile saline solution and stored at −80 °C.

### 4.3. DNA Extraction and Library Preparation

After being thawed, samples were vortexed, and then total DNA was extracted from 300 µL of each sample in a final elution volume of 50 µL by the automatic extractor Maxwell CSC DNA Blood Kit (Promega, Madison, WI, USA), according to the manufacturer’s instructions.

The bacterial composition of the samples was performed by sequencing region V3 of the 16S rRNA gene. Briefly, a qPCR targeting the V1-V3 region of the 16S rRNA gene (500 bp) was performed by employing the U534R primer and the degenerated primer 27FYM. Subsequently, a semi-nested PCR was carried out with the primers B338F_P1-adaptor and U534R_A_adaptor_barcode, targeting the V3 region (200 bp) of the 16S rRNA gene, with a different barcode for each sample linked to the reverse primer [45]. The PCR reactions were performed using EvaGreen^®^ dye (Fisher Molecular Biology, Waltham, MA, USA), the Kapa 2G HiFi Hotstart ready mix 2X (Kapa Biosystems, Wilmington, MA, USA), 0.5 µM of each primer, and 400 ng/µL of Bovine Serum Albumin (BSA), in a final volume of 10 µL. The temperature cycling conditions were: 5 min at 95 °C, 30 s at 95 °C, 30 s at 59°/57 °C, 45 s at 72 °C and a final elongation step at 72 °C for 10 min. No template controls, processed in parallel with clinical samples starting from the pre-analytic phase, were used as negative controls. The correct size of the amplicons (560 bp for the first PCR and 260 bp for semi-nested PCR) was assessed on a 2% agarose gel. The amount of dsDNA in each sample after the semi-nested PCR was quantified with a Qubit^®^ 2.0 Fluorimeter (Invitrogen, Carlsbad, California, USA) using the Qubit^®^ dsDNA BR Assay Kit (Thermo Fisher Scientific, Waltham, MA, USA), and an equal amount of each sample (100 ng) was mixed into a single batch to generate a pooled library at a final concentration of 100 pM, according to the manufacturer’s instructions. Template preparation was performed by emulsion PCR using the Ion OneTouch™ 2 System (Life Technologies, Gran Island, New York, NY, USA), with the Ion PGM Hi-Q View OT2 200 kit (Life Technologies, New York, NY, USA), and subsequent quality control was carried out on a Qubit^®^ 2.0 Fluorimeter. Sequencing was performed with the Ion PGM™ System technology by using the Ion PGM Hi-Q View sequencing kit (Life Technologies, New York, NY, USA).

### 4.4. Dosage of Immune Factors

The soluble concentration of 27 cytokines, chemokines, and growth factors was assessed in duplicate in all 60 vaginal swabs, amniotic fluid, and chorionic villi samples using magnetic bead-based multiplex immunoassays (Bioplex ProTM human cytokine 27-plex panel, Bio-Rad Laboratories, Milan, Italy), according to the pre-optimized protocol [46]. Regarding vaginal swabs, after centrifugation at 1000× *g*, the undiluted samples (50 μL) were mixed with biomagnetic beads in 96-well flat-bottom plates with the addition of 0.5% of BSA. The amniotic fluid and chorionic villi samples were centrifugated at 1000× *g*, and diluted 1:4 using Bioplex Sample Diluent before analysis. Then, 50 μL of diluted samples were mixed with biomagnetic beads in 96-well flat-bottom plates. After incubation for 30 min at room temperature, followed by a washing plate with Bio-Plex wash buffer, 25 μL of the antibody–biotin reporter was added. After the addition of 50 μL of streptavidin–phycoerythrin and following incubation for 10 min, the concentrations of the cytokines were determined using the Bio-Plex-200 system (Bio-Rad Corp., United States) and Bio-Plex Manager software (v.6, Bio-Rad). The data were expressed as median fluorescence intensity (MFI) and concentration (pg/mL).

### 4.5. Statistical Analysis

For the big data analysis, using QIIME 2.22.2, evenness (how equally distributed are the species within a community; value = 1 when all species have the same abundance) and observed ASVs (the total number of species in the samples) metrics were calculated to assess the alpha diversity (microbiome diversity within a community) and compared by means of the Kruskal–Wallis test. The Bray–Curtis dissimilarity index was calculated to assess the beta diversity, which measures the similarity or dissimilarity of the analyzed groups, visualized by the principal coordinate analysis (PcoA), and compared by the PERMANOVA test. To highlight the differences in the microbial composition, we performed differential abundance testing using the ANCOM test. Using MicrobiomeAnalyst, we applied the LEfSE test to identify microbiological biomarkers [47].

To test the differences in the immune-soluble factors, GraphPad Prism (v. 5, San Diego, CA, USA) was used. Specifically, the Kruskal–Wallis one-way analysis of variance was used for comparisons between groups. When a significant *p*-value was observed (*p* < 0.05), a multiple comparison test was used to determine which groups were different.

### 4.6. Data Availability

Due to the sensitive nature of the data, information created during and/or analyzed during the current study is available from the corresponding author upon reasonable request to *bona fide* researchers.

### 4.7. Ethical Statement

All the participants signed an informed consent, and the experiment was carried out according to the principles of the Declaration of Helsinki. Ethical approval for this study was obtained from the ethical committee board (CEUR-2019-sper-154, protocol number 0037797).

## 5. Conclusions

Taken together, our results support the presence of bacterial DNA in CVS and AF samples. These results are relevant as the exposure of the fetus to the maternal microbiome could impact the proper development of the fetal immune system but also play a vital role in predisposing the newborn to pathologies such as asthma, allergies, obesity, neurodevelopmental disorders, and liver steatosis [48,49].

Further studies on a wider cohort are needed to understand the possible role of bacterial colonization of amniotic fluid and chorionic villi, the associated immune response in the pregnancy outcome, and the route of colonization. In this regard, studies should highlight if a hematogenous spread could justify the spread of bacterial DNA belonging to oral microbes to the placenta. If, in the vaginal microbiome, the dysbiosis could contribute to the likelihood of retrieving CVS and AF samples positive for bacterial DNA. If, despite the absence of an ongoing infection, bacterial translocation could be elicited or accompanied by the upregulation of inflammatory cytokines in the amniotic fluid coupled with an immune hyporesponsiveness in the vaginal milieu.

On one side, we had the possibility to collect CVS to study a microbial composition that is less explored compared to AF samples and placentas of at-term pregnancies. In addition, we simultaneously collected samples from the other maternal body sites in order to identify the possible origin of the DNA identified in the CVS and AF samples. On the other side, we acknowledge that our cohort is small, and we cannot argue for the clinical relevance of our findings. Furthermore, we cannot demonstrate the route of colonization of CVS and AF samples from maternal body districts.

## Data Availability

Due to the sensitive nature of the data, information created during and/or analyzed during the current study is available from the corresponding author upon reasonable request to bona fide researchers.

## Supplementary Materials

The Supplementary Materials are available online at https://www.mdpi.com/article/10.3390/ijms24032873/s1.
