# Supplementary material for: The Bacterial DNA Profiling of Chorionic Villi and Amniotic Fluids Reveals Overlaps with Maternal Oral, Vaginal, and Gut Microbiomes
Source: Int J Mol Sci. 2023 Feb 2;24(3):2873. doi: 10.3390/ijms24032873 (PMC9917689; doi:10.3390/ijms24032873)
Supplement: Supplementary file 1 [file ijms-24-02873-s001.zip › ijms-2134770-supplementary.pdf]

Table S1. The kitome. Unique bacteria identified in one or more no template controls (n = 24) and identified in less than 30% of these samples. The names of bacteria are reported, and several features correspond to each bacterium.

**Bacteria**
Stenotrophomonas spp.
Methylobacterium-Methylorubrum spp.
Chryseobacterium spp.
Cloacibacterium spp.
Empedobacter spp.
Pedobacter spp.
Poterioochromonas spp.
Desulfovibrio spp.
Anaerobacillus spp.
Exiguobacterium spp.
Weissella paramesenteroides
Saccharimonadales spp.
Azospirillum spp.
Brevundimonas spp.
Devosia spp.
Novosphingobium spp.
Sphingomonas spp.
Delftia spp.
Diaphorobacter spp.
Herbaspirillum spp.
Dechloromonas spp.
Enhydrobacter spp.

Table S2. PERMANOVA. Results of the pairwise PERMANOVA of the Bray–Curtis dissimilarity matrix. CVS = chorionic villus samples; Vag.CVS = vaginal swabs matched to CVS; Rect.CVS = rectal swabs matched to CVS samples; Sal.CVS = saliva samples matched to CVS; AF = amniotic fluid samples; Vag.AF = vaginal swabs matched to AF; Rect.AF = rectal swabs matched to AF samples; Sal.AF = saliva samples matched to AF.

**PAIRWISE PERMANOVA**			
**CVS vs**		**Pseudo-F**	FDR *p* value
	**Vag.CVS**	1.6	0.03
	**Rect.CVS**	2	0.001
	**Sal.CVS**	3.6	0.001
**AF vs**		**Pseudo-F**	FDR *p* value
	**Vag.AF**	2.8	0.001
	**Rect.AF**	2.3	0.003
	**Sal.AF**	3.9	0.001

Table S3. Biomarkers at the genus level. Biomarkers identified by the LEfSe test at the genus level in the vaginal, rectal, and saliva samples matched to the negative CVS/AF samples compared with those matched to the positive CVS/AF samples.

**Biomarker**	**LDA Score**	**FDR *p* Value**
Alloprevotella	5.15	<0.001
Campylobacter	6.02	<0.001
Dialister	5.66	0.001
Fusobacterium	5.65	<0.001
Gemella	5.46	<0.001
Granulicatella	5.66	<0.001
Haemophilus	5.85	<0.001
Lactobacillus	6.68	<0.001
Peptoniphilus	5.97	<0.001
Prevotella	6.04	0.002
Rothia	5.03	<0.001
Staphylococcus	5.74	<0.001
Streptococcus	6.37	<0.001
TM7x	5.19	<0.001
Veillonella	5.94	<0.001

Table S4. Biomarkers at the species level. Biomarkers identified by the LefSe test at the species level in the vaginal, rectal, and saliva samples matched to the negative CVS/AF samples compared with those matched to the positive CVS/AF samples.

Biomarker	LDA Score	FDR *p* Value
Campylobacter ureolyticus	6.02	<0.001
Lactobacillus crispatus	6.44	<0.001
Prevotella bivia	5.94	<0.001
Staphylococcus epidermidis	5.74	<0.001
Streptococcus salivarius	5.62	<0.001
